# Supplementary material for: Spatial transcriptomics using multiplexed deterministic barcoding in tissue
Source: Nat Commun. 2023 Mar 18;14:1523. doi: 10.1038/s41467-023-37111-w (PMC10024691; doi:10.1038/s41467-023-37111-w)
Supplement: Supplementary file 1 — Supplementary Information [file 41467_2023_37111_MOESM1_ESM.pdf]

Supplemental information

Spatial Transcriptomics Using Multiplexed Deterministic Barcoding in Tissue

Johannes Wirth[1], Nina Huber[1], Kelvin Yin[1], Sophie Brood[1], Simon Chang[1], Celia P. Martinez-Jimenez[1,2,*], Matthias Meier[1,3,*]

[1]Helmholtz Pioneer Campus, Helmholtz Munich, Munich, Germany

[2]TUM School of Medicine, Technical University of Munich, Munich, Germany

[3]Center for Biotechnology and Biomedicine, University of Leipzig, Leipzig, Germany

*Corresponding authors: celia.martinez@helmholtz-muenchen.de, matthias.meier@helmholtz-muenchen.de

Supplemental Figures

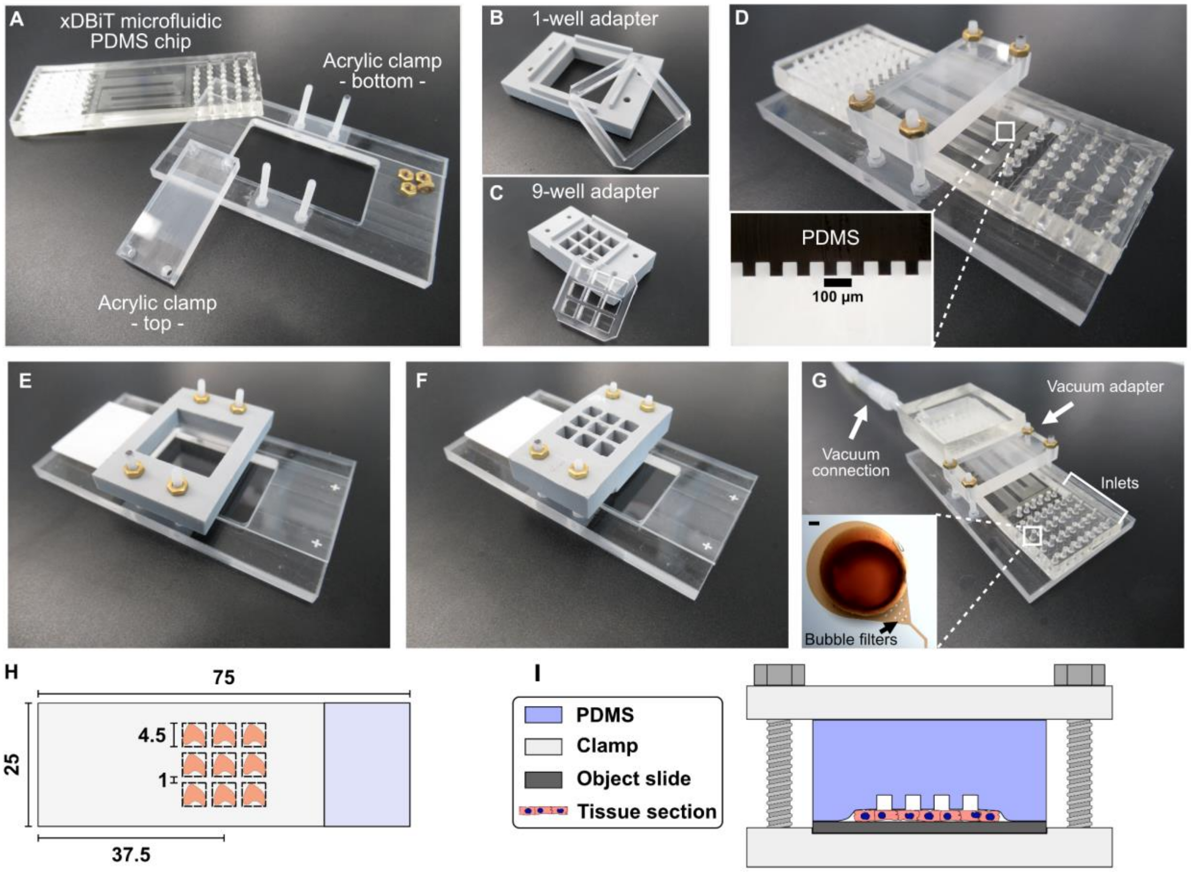

Supplementary Figure 1. Overview of xDBiT components. (A) All components required to perform spatial barcoding using the xDBiT microfluidic PDMS chip. **(B)** Image of 3D-printed 1-well adapter and respective PDMS gasket to treat all tissue sections at once. **(C)** Image of 3D-printed 9-well adapter and respective PDMS gasket used to perform the reverse transcriptions and lysis steps. **(D)** xDBiT microfluidic PDMS chip with assembled clamp. Detail image shows cross section of microfluidic channels with heights and widths of 50 µm. Scale bar: 100 µm. **(E and F)** Assembled 1-well and 9-well adapters with clamp, respectively. **(G)** Assembled xDBiT microfluidic PDMS chip, clamps, and attached vacuum adapter. Enlarged image shows one inlet filled with food dye-colored water to illustrate the bubble filters at the transition of inlet to channel. Scale bar: 100 µm. **(H)** Schematic showing the positioning of tissue sections compatible with xDBiT. **(I)** Schematic cross section of a xDBiT PDMS chip and clamp system.

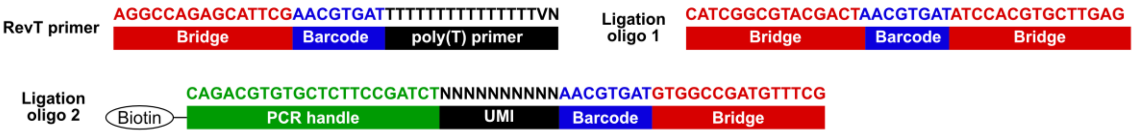

Supplementary Figure 2. Sequences of barcoding oligonucleotides used during xDBiT workflow. RevT: Reverse transcription; UMI: Unique molecular identifier; PCR: Polymerase chain reaction.

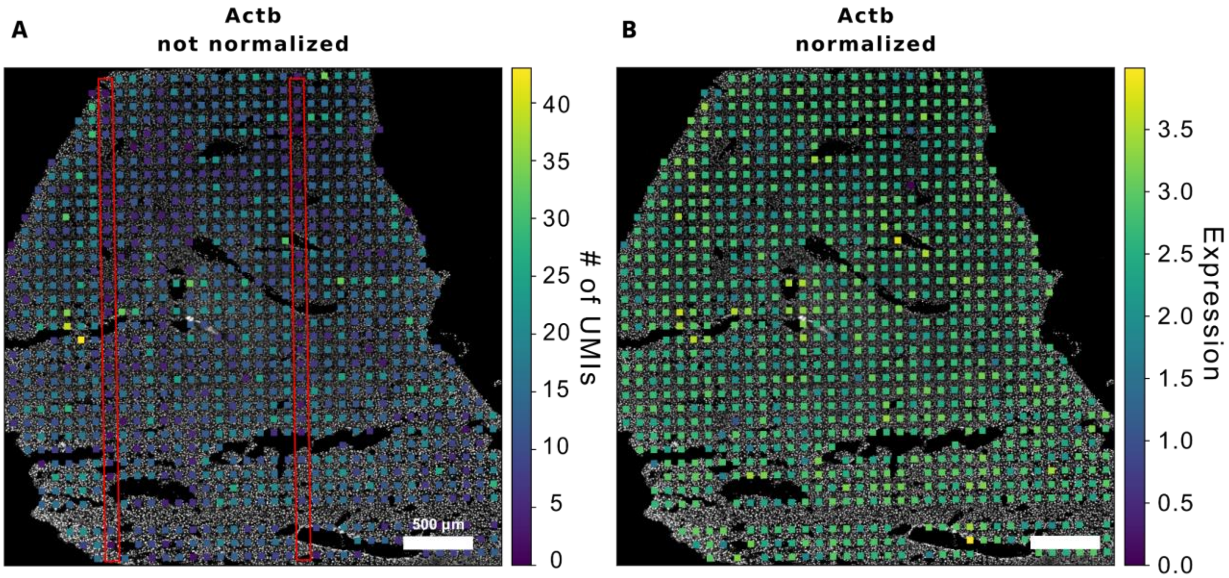

Supplementary Figure 3. Strip artifact in xDBiT data. Spatial projection of different xDBiT results on DAPI image of the tissue section to investigate the occurrence of stripe artifacts. The spot color corresponds to the number of *Actb* UMIs before normalization **(A)**, or the normalized and log-transformed expression of *Actb* **(B)**. The red box indicates strip effects observed during experiments.

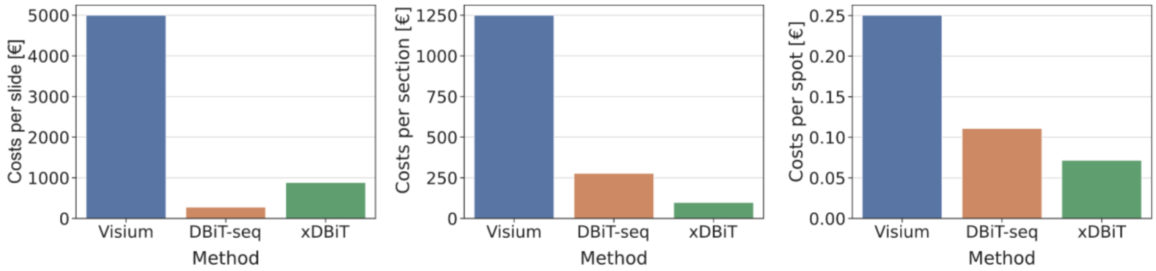

Supplementary Figure 4. Comparison of xDBiT costs with Visium Spatial [1] and DBiT-seq method [2]. The panels from left to right show the costs normalized per slide, section and spot, respectively. For DBiT-seq we calculated the costs from one experiment performed in our lab.

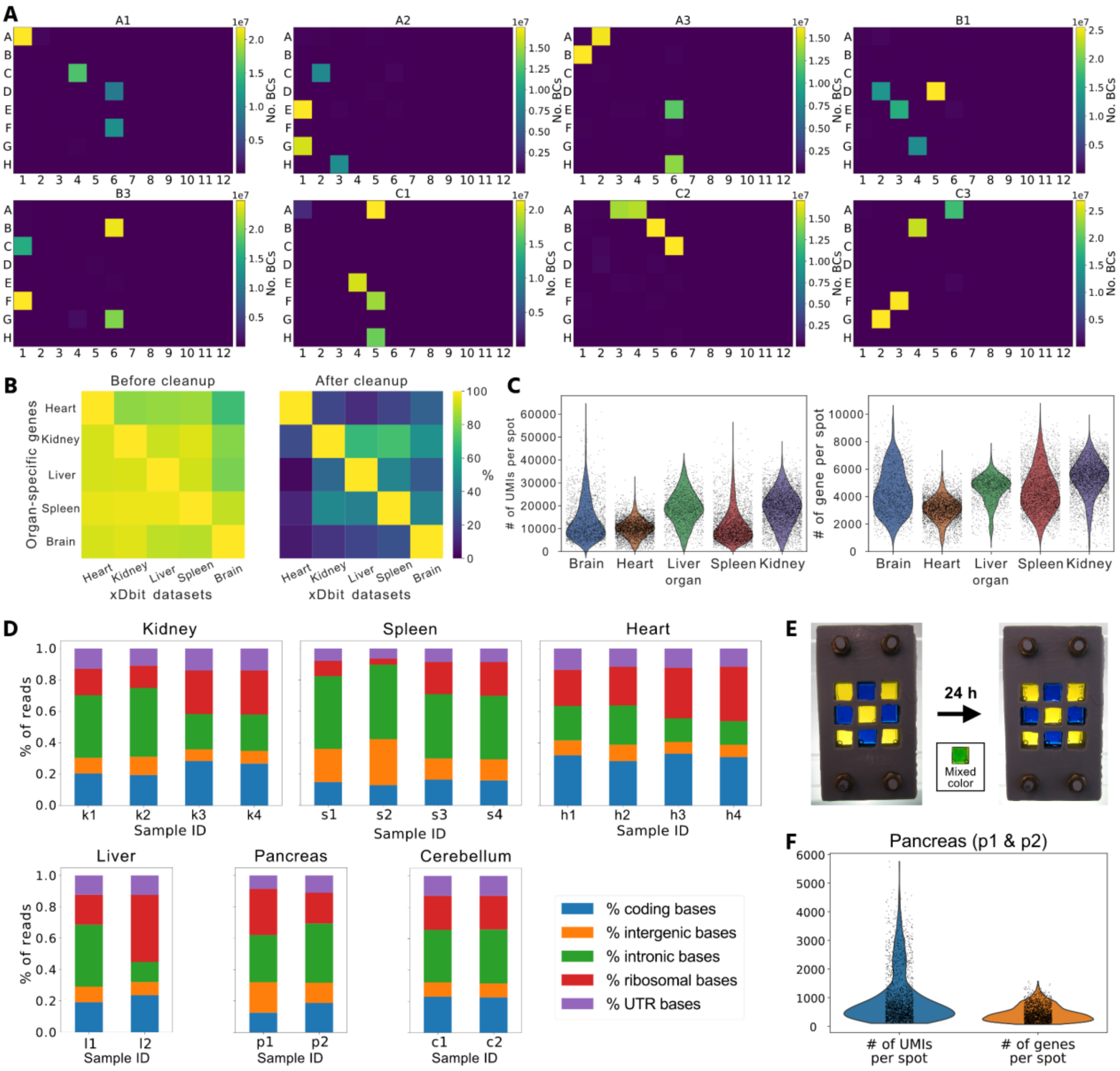

Supplementary Figure 5. Quality control analyses for xDBit. (A) Illustration of plate position of barcodes found in the different wells during cross-contamination analysis. **(B)** Percentage of organ-specific genes that were found in the xDBiT datasets before and after computational background removal. Organ-specific genes were taken from HOMER database[3]. **(C)** Number of UMIs and genes per spot before computational removal of background genes. **(D)** Shown are the alignment coverage metrics for all experiments published in this study grouped by organ of origin. For information about the sample IDs see **Supp. Table 2**. **(E)** Test of cross-contamination in the 9-well adapter using food dye-colored water. Images taken before and after 24 h incubation. Box shows example image of the color resulting from mixing yellow and blue. **(F)** Count statistics in the pancreas samples. Violin plot shows numbers of UMIs per spot and number of genes per spot before filtering. UMI: Unique molecular identifier; c: Cerebellum, h: Heart, l: Liver, s: Spleen, k: Kidney, p: Pancreas, numbers indicate replicates.

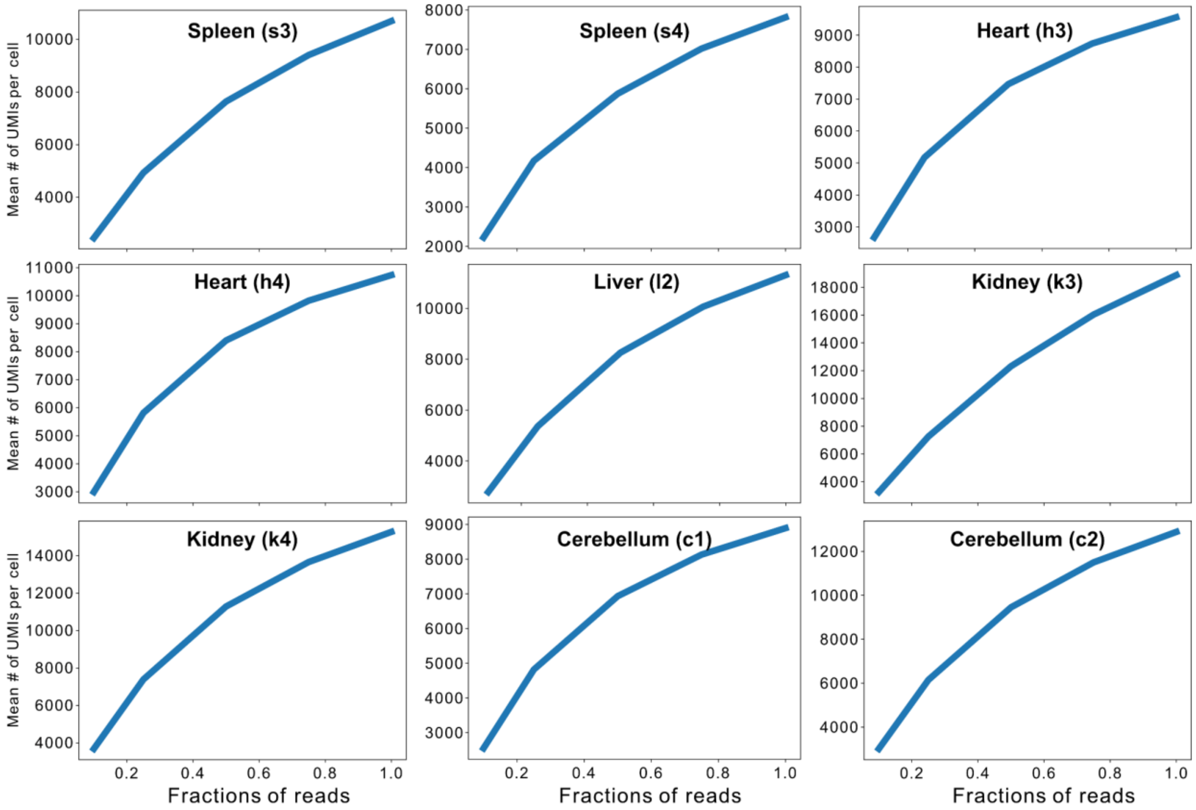

Supplementary Figure 6. Sequencing read saturation in xDBiT. Analysis of sequencing saturation by random subsampling of reads before count matrix generation. Line plots show the mean number of UMIs per spot retrieved at different fractions of reads used for subsampling. The brackets contain the sample ID. For more information on the sample IDs see **Supp. Table 2**.

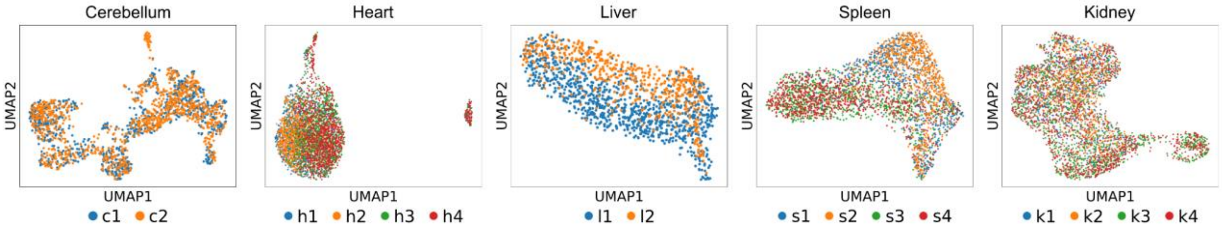

Supplementary Figure 7. Visualizing batch effects in the multi-organ datasets. Dimensionality reduction was performed using the UMAP algorithm[4] and colors denote the individual tissue sections. UMAP: Uniform Manifold Approximation and Projection; c: Cerebellum, h: Heart, l: Liver, s: Spleen, k: Kidney, numbers indicate replicates.

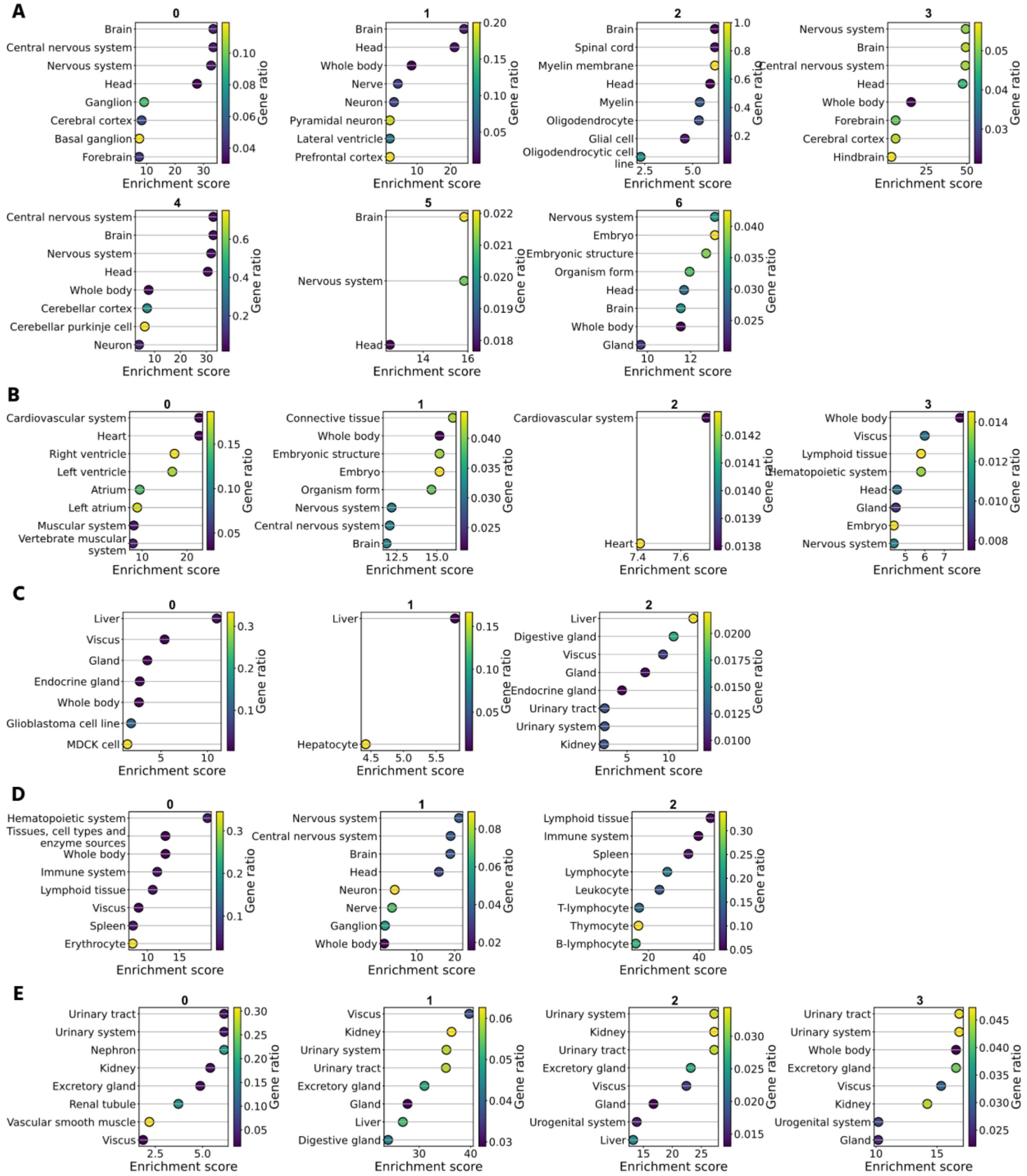

Supplementary Figure 8. Gene ontology (GO) term enrichment analysis of *Leiden* clusters. GO term enrichment analysis was performed using the STRING algorithm[5] and the *Brenda Tissue Ontology* database [6] for all Leiden clusters in cerebellum **(A)**, heart **(B)**, liver **(C)**, spleen **(D)**, and kidney **(E)**. P-values for each GO term were calculated using a Hypergeometric test and corrected for multiple testing using the Benjamini-Hochberg procedure[7]. The enrichment score was calculated as negative logarithm of the corrected p-value. The gene ratio describes the size of the enrichment effect as logarithmic ratio of observed and expected number of genes.

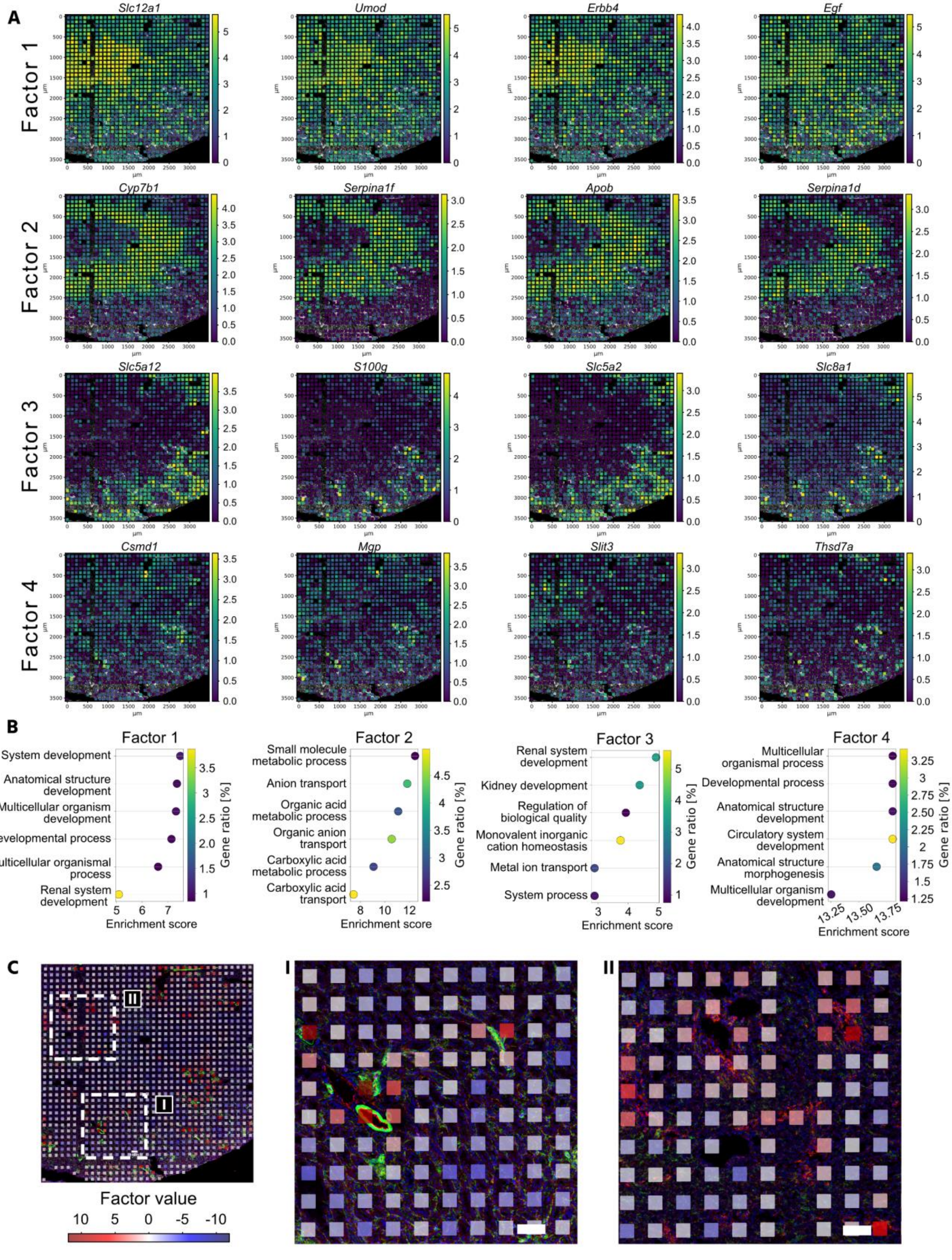
A
Factor 1
Slc12a1
Umod
Erbb4
Egf
Factor 2
Cyp7b1
Serpina1f
Apob
Serpina1d
Factor 3
Slc5a12
S100g
Slc5a2
Slc8a1
Factor 4
Csmd1
Mgp
Slit3
Thsd7a
µm
B
Factor 1
System development
Anatomical structure development
Multicellular organism development
Developmental process
Multicellular organismal process
Renal system development
Enrichment score
Gene ratio [%]
Factor 2
Small molecule metabolic process
Anion transport
Organic acid metabolic process
Organic anion transport
Carboxylic acid metabolic process
Carboxylic acid transport
Factor 3
Renal system development
Kidney development
Regulation of biological quality
Monovalent inorganic cation homeostasis
Metal ion transport
System process
Factor 4
Multicellular organismal process
Developmental process
Anatomical structure development
Circulatory system development
Anatomical structure morphogenesis
Multicellular organism development
C
I
II
Factor value
10 5 0 -5 -10

Supplementary Figure 9. Extended factor analysis of one exemplary kidney section. Factor analysis was performed using MEFISTO [8]. **(A)** Top positively weighted genes for the first 4 factors projected onto the respective phalloidin flourescence image. Colors denote the expression of the gene in the respective xDBiT spot. **(B)** GO term enrichment analysis using the STRING algorithm[5] and the *Biological Processes* GO database[9,10]. As input we used the top positively weighted genes (> 95% confidence interval) of the first four factors to reveal functional and structural areas of the murine kidney. P-values for each GO term were calculated using a Hypergeometric test and corrected for multiple testing using the Benjamini-Hochberg procedure[7]. The enrichment score was calculated as negative logarithm of the corrected p-value. The gene ratio describes the size of the enrichment effect as logarithmic ratio of observed and expected number of genes. **(C)** Details of the projection of MEFISTO factor 4 onto fluorescence images of the kidney section at two exemplary positions (I and II). Image colors denote CD31 (red), phalloidin (green) and DAPI (blue). The images show a correlation of the endothelial marker CD31 with factor 4. Scale bar: 100 µm.

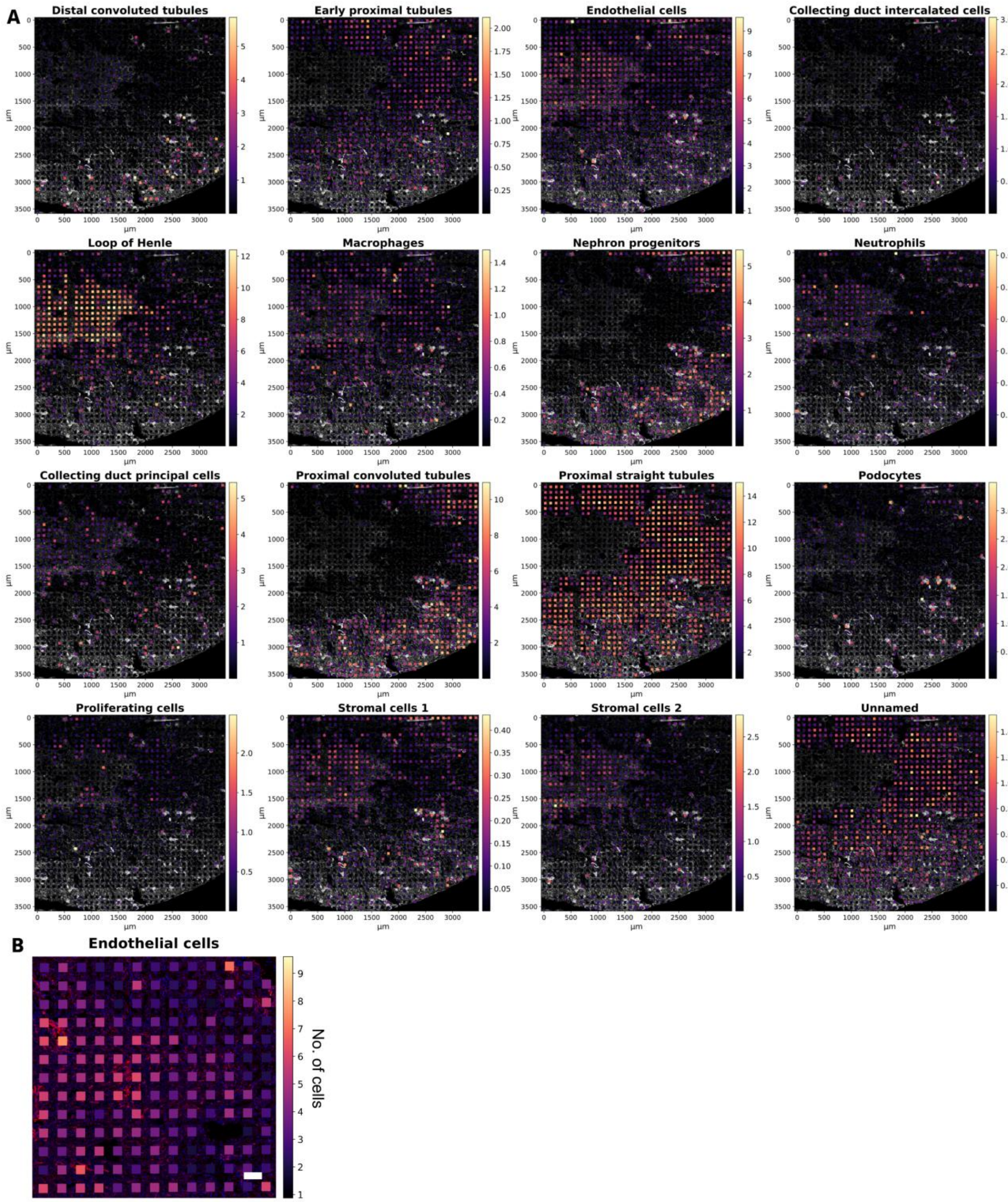

Supplementary Figure 10. Extended cell type mapping results to the representative kidney section. Deconvolution of the xDBiT ST results was performed using *cell2location* [11] and a published single-cell transcriptomics (scT) dataset of mural kidneys [12]. **(A)** Spatially mapped proportions of all cell types, predicted by cell2location, onto the phalloidin fluorescence image of the kidney section. Spot colors denote the minimum number of cells predicted. **(B)** Detail of a representative region of the deconvolution results for endothelial cells. Spot colors denote the minimum number of predicted cells.

Supplementary Tables

	1	2	3	4	5
A		9	17	25	
B	2	10	18	26	34
C	3	11	19	27	35
D	4	12	20	28	36
E	5	13	21	29	37
F	6	14	22	30	38
G	7	15	23	31	39
H		16	24	32	

Alignment marker
Barcode #

Supplementary Table 1. Filling scheme of inlets for barcoding rounds with the xDBiT microfluidic PDMS chip. Inlets filled with alignment markers and ligation barcodes are colored red and green, respectively.

Experiment	Organ	Sample ID	xDBiT run ID	Well positions	Mouse ID
**Multi-organ**	Heart	h1	1	A3	TS26
	Heart	h2	1	B1	TS26
	Kidney	k1	1	B3	TS26
	Kidney	k2	1	C1	TS26
	Liver	l1	1	B2	TS26
	Spleen	s1	1	A1	TS26
	Spleen	s2	1	A2	TS26
	Pancreas	p1	1	C2	TS27
	Pancreas	p2	1	C3	TS27
	Heart	h3	2	A3	TS27
	Heart	h4	2	B1	TS27
	Kidney	k3	2	B3	TS27
	Kidney	k4	2	C1	TS27
	Liver	l2	2	B2	TS27
	Spleen	s3	2	A1	TS27
	Spleen	s4	2	A2	TS27
	Cerebellum	c1	2	C2	NaCai1
	Cerebellum	c2	2	C3	NaCai1
**Cross-contamination analysis**	Liver	l3	3	A1	TS16
	Liver	l4	3	A2	TS16
	Liver	l5	3	A3	TS16
	Liver	l6	3	B1	TS16
	Liver	l7	3	B3	TS5
	Liver	l8	3	C1	TS5
	Liver	l9	3	C2	TS5
	Liver	l10	3	C3	TS5

Supplementary Table 2. Information about the origin of the samples used in this study. Every xDBiT run ID refers to one experiment in which one xDBiT PDMS chip was used. The well positions refer to the position on the 3 x 3 grid on a PDMS chip.

Organ	Encode ID	Assay type	# of isogenic replicates
Heart	ENCSR000CGZ	polyA plus RNA-seq	2
Kidney	ENCSR000CHA	polyA plus RNA-seq	2
Spleen	ENCSR000CGW	polyA plus RNA-seq	2
Spleen	ENCSR966JPL	polyA plus RNA-seq	2
Liver	ENCSR000CHB	polyA plus RNA-seq	2
Cerebellum	ENCSR000CGX	polyA plus RNA-seq	2

Supplementary Table 3. Information about bulk RNA-seq datasets used in this study. The datasets were downloaded from the ENCODE[13,14] database.

		RevT Barcode								
	%	A1	A2	A3	B1	B3	C1	C2	C3	Spillover
Well	A1	*91.89*	1.26	1.69	1.45	1.06	0.96	0.78	0.91	8.11
	A2	1.31	*90.64*	1.46	1.61	1.17	1.32	1.48	1.01	9.36
	A3	1.28	1.02	*92.50*	1.23	1.33	1.07	0.77	0.81	7.50
	B1	1.35	1.20	2.35	*90.79*	1.23	1.12	0.97	0.99	9.21
	B3	0.88	0.85	1.25	1.88	*92.40*	0.97	0.67	1.11	7.60
	C1	3.50	0.54	0.72	0.62	0.64	*92.66*	0.63	0.69	7.34
	C2	1.05	1.22	1.32	1.85	1.09	1.10	*91.35*	1.02	8.65
	C3	0.81	0.62	1.31	0.87	0.79	0.77	0.53	*94.31*	5.69

Supplementary Table 4. Results of the spillover analysis. The table shows the percentage of reads in a certain well (rows) that was found to carry a certain RevT barcode (columns). Italic values mark the combinations of well and barcode that were expected from the experimental setup. All other values are derived from spillover events between the wells. The right column shows the sum of spillover events per well. RevT: Reverse transcription.

Cell type	Cell2location [%]	Clark et al., 2019 [%]
**Distal convoluted tubules**	1.91	7.45
**Early proximal tubules**	2.29	
**Endothelial cells**	17.27	
**Collecting duct intercalated cells**	0.68	6.96 (All A-ICs + B-ICs)
**Loop of Henle**	15.70	14.87 (mTAL)
**Macrophages**	1.54	
**Nephron progenitors**	4.40	
**Neutrophils**	0.38	
**Collecting duct principal cells**	2.87	4.96 (CCD PC + OMCD PC)
**Proximal convoluted tubules**	10.77	44
**Proximal straight tubules**	34.55	
**Podocytes**	1.67	3
**Proliferating cells**	1.12	
**Stromal cells 1**	0.47	
**Stromal cells 2**	2.01	
**Unnamed**	2.37	

Supplementary Table 5. Cell type percentages obtained from the cell2location analysis and subsequential comparsion to published percentages from[15]. Medullary thick ascending loop (mTAL), principal cells (PCs), Cortical collecting duct (CCD), Outer medullary collecting duct (OMCD), Intercalated cells type A and B (A-ICs + B-ICs).

Supplementary References

1. Salmén, F. *et al.* Barcoded solid-phase RNA capture for Spatial Transcriptomics profiling in mammalian tissue sections. *Nat. Protoc.* **13**, 2501–2534 (2018).
2. Liu, Y. *et al.* High-Spatial-Resolution Multi-Omics Sequencing via Deterministic Barcoding in Tissue. *Cell* **183**, 1665-1681.e18 (2020).
3. Zhang, F. & Chen, J. Y. HOMER: a human organ-specific molecular electronic repository. *BMC Bioinformatics* **12**, S4 (2011).
4. McInnes, L., Healy, J. & Melville, J. UMAP: Uniform Manifold Approximation and Projection for Dimension Reduction. (2018).
5. Szklarczyk, D. *et al.* STRING v11: protein–protein association networks with increased coverage, supporting functional discovery in genome-wide experimental datasets. *Nucleic Acids Res.* **47**, D607–D613 (2019).
6. Gremse, M. *et al.* The BRENDA Tissue Ontology (BTO): the first all-integrating ontology of all organisms for enzyme sources. *Nucleic Acids Res.* **39**, D507–D513 (2011).
7. Franceschini, A. *et al.* STRING v9.1: protein-protein interaction networks, with increased coverage and integration. *Nucleic Acids Res.* **41**, D808–D815 (2013).
8. Velten, B. *et al.* Identifying temporal and spatial patterns of variation from multimodal data using MEFISTO. *Nat. Methods* 1–8 (2022) doi:10.1038/s41592-021-01343-9.
9. The Gene Ontology Consortium. The Gene Ontology resource: enriching a GOld mine. *Nucleic Acids Res* (2021) doi:10.1093/nar/gkaa1113.
10. Ashburner, M. *et al.* Gene ontology: Tool for the unification of biology. *Nat. Genet.* **25**, 25–29 (2000).
11. Kleshchevnikov, V. *et al.* Cell2location maps fine-grained cell types in spatial transcriptomics. *Nat. Biotechnol.* 1–11 (2022) doi:10.1038/s41587-021-01139-4.

12. Miao, Z. *et al.* Single cell regulatory landscape of the mouse kidney highlights cellular differentiation programs and disease targets. *Nat. Commun.* **12**, 1–17 (2021).
13. Davis, C. A. *et al.* The Encyclopedia of DNA elements (ENCODE): data portal update. *Nucleic Acids Res.* **46**, D794–D801 (2018).
14. Dunham, I. *et al.* An integrated encyclopedia of DNA elements in the human genome. *Nature* **489**, 57–74 (2012).
15. Clark, J. Z. *et al.* Representation and relative abundance of cell-type selective markers in whole-kidney RNA-Seq data. *Kidney Int.* **95**, 787–796 (2019).
