# Supplementary material for: Spatial transcriptomics using multiplexed deterministic barcoding in tissue
Source: Nat Commun. 2023 Mar 18;14:1523. doi: 10.1038/s41467-023-37111-w (PMC10024691; doi:10.1038/s41467-023-37111-w)
Supplement: Supplementary file 3 — Description of Additional Supplementary Files [file 41467_2023_37111_MOESM3_ESM.pdf]

Description of Additional Supplementary Files

File Name: Supplementary Data 1
Description: Sequences of primers for reverse transcription with respective 5' modifications.

File Name: Supplementary Data 2
Description: Sets of reverse transcription primer barcodes to encode the nine tissue sections. Optimal mixtures were determined using BARCOSEL.

File Name: Supplementary Data 3
Description: Sequences of ligation round 1 oligonucleotides with their respective 5' modifications.

File Name: Supplementary Data 4
Description: Sequences of ligation round 2 oligonucleotides with their respective 5' modifications.

File Name: Supplementary Data 5
Description: Sequences of the bridge oligonucleotides and primers used during xDBiT library preparation.
