# Supplementary material for: Spatial transcriptomics using multiplexed deterministic barcoding in tissue
Source: Nat Commun. 2023 Mar 18;14:1523. doi: 10.1038/s41467-023-37111-w (PMC10024691; doi:10.1038/s41467-023-37111-w)
Supplement: Supplementary file 9 — Reporting Summary [file 41467_2023_37111_MOESM9_ESM.pdf]

nature portfolio

Corresponding author(s): Celia P. Martinez-Jimenez and Matthias Meier

Last updated by author(s): Feb 10, 2023

Reporting Summary

Nature Portfolio wishes to improve the reproducibility of the work that we publish. This form provides structure for consistency and transparency in reporting. For further information on Nature Portfolio policies, see our Editorial Policies and the Editorial Policy Checklist.

Statistics

For all statistical analyses, confirm that the following items are present in the figure legend, table legend, main text, or Methods section.

n/a	Confirmed	
☐	☒	The exact sample size (*n*) for each experimental group/condition, given as a discrete number and unit of measurement
☐	☒	A statement on whether measurements were taken from distinct samples or whether the same sample was measured repeatedly
☐	☒	The statistical test(s) used AND whether they are one- or two-sided<br>*Only common tests should be described solely by name; describe more complex techniques in the Methods section.*
☐	☒	A description of all covariates tested
☐	☒	A description of any assumptions or corrections, such as tests of normality and adjustment for multiple comparisons
☐	☒	A full description of the statistical parameters including central tendency (e.g. means) or other basic estimates (e.g. regression coefficient) AND variation (e.g. standard deviation) or associated estimates of uncertainty (e.g. confidence intervals)
☐	☒	For null hypothesis testing, the test statistic (e.g. *F*, *t*, *r*) with confidence intervals, effect sizes, degrees of freedom and *P* value noted<br>*Give P values as exact values whenever suitable.*
☒	☐	For Bayesian analysis, information on the choice of priors and Markov chain Monte Carlo settings
☒	☐	For hierarchical and complex designs, identification of the appropriate level for tests and full reporting of outcomes
☐	☒	Estimates of effect sizes (e.g. Cohen's *d*, Pearson's *r*), indicating how they were calculated

Our web collection on statistics for biologists contains articles on many of the points above.

Software and code

Policy information about availability of computer code

Data collection	Imaging was performed using a Zeiss LSM 880 and the ZEN 2.3 SP1 FP3 (black) software.<br>Sequencing was done using an Illumina NovaSeq 6000 system .
Data analysis	Upstream analysis of transcriptomic data:<br>All custom code and pipelines to analyze the raw sequencing data can be found under "ReadsToCounts" and "CountsToAnndata" in https://github.com/jwrth/xDbit_toolbox. The pipeline is based on Drop-seq_tools-2.1.0 and the Split-seq pipeline provided under: https://github.com/RebekkaWegmann/splitseq_toolbox.<br><br>The pipelines use following software and packages:<br>- For genomic alignment we used STAR (2.7.4a).<br>- For image processing, transformation and registration Fiji ImageJ (v1.53c), OpenCV (4.6.0) and napari (0.4.5) were used.<br>- The SIFT algorithm is used to extract common features between the alignment DAPI image and the high-resolution DAPI image.<br>- Read trimming was performed with cutadapt v3.7.<br>- SAM/BAM files were processed with samtools v1.9 and the Python package pysam (v0.19.1).<br>- Further, the pipeline includes functions from Squidpy (v1.1.2).<br><br>Downstream analysis of transcriptomic data:<br>- For general spatial transcriptomic preprocessing, data analysis and plotting, functions from Scanpy (v1.8.2), Squidpy (v1.1.2). and custom functions provided in https://github.com/jwrth/xDbit_toolbox/xdbit_funcs were used.<br>- Batch correction was performed per section using scanorama (v1.7.2).<br>- For GO term enrichment analysis the STRING database and the gprofiler web resources were used.

- For MEFISTO factor analysis the Python package mofapy2 (v0.6.4) was used.
- Deconvolution of spatial transcriptomic datasets with single-cell RNA-seq datasets was performed using cell2location (v0.1). In the deconvolution process, anndatas were prepared for analysis using scvi-tools (v0.16.4).
- Linear regression was performed using the Python package statsmodels (v0.12.2).
- For plotting the Python packages matplotlib v3.5.1 and seaborn v0.11.2 were used.

Image analysis:
- For general image processing and generation of figures, we used Fiji ImageJ v1.53c and the Quickfigures toolkit.
- Stitching of the tiled images was performed using a custom ImageJ script utilizing the Grid/Collection Stitching algorithm.

Experimental preparations:
- The BARCOSEL tool was used to generated optimal reverse transcription primer mixes.

For manuscripts utilizing custom algorithms or software that are central to the research but not yet described in published literature, software must be made available to editors and reviewers. We strongly encourage code deposition in a community repository (e.g. GitHub). See the Nature Portfolio guidelines for submitting code & software for further information.

Data

Policy information about availability of data

All manuscripts must include a data availability statement. This statement should provide the following information, where applicable:

- Accession codes, unique identifiers, or web links for publicly available datasets
- A description of any restrictions on data availability
- For clinical datasets or third party data, please ensure that the statement adheres to our policy

Sequencing data:
All raw sequencing data and preprocessed xDBiT data including spatial transcriptomic data with aligned images are available on GEO under the accession number GSE207843 (Reviewer token: cjkxueuwddanhcp).

Third party resources:
- For our analysis we searched for enrichments in the Brenda Tissue Ontology database (BTO) and the Biological Processes GO database.
- Information about protein expression of differentially expressed genes in the respective tissues has been taken from The Human Protein Atlas.
- Previously published DBiT-seq datasets from embryonic sections were downloaded from the Gene Expression Omnibus database with the accession code GSE137986. Of the whole dataset following experiments were retrieved for the comparison: GSM4189613 (Embryo stage E10 – 162,684,631 raw reads) and GSM4189612 (Embryo stage E12 – 53,619,846 raw reads).
- Bulk polyA plus RNA-seq data was obtained from ENCODE database: ENCSR000CGZ (Heart), ENCSR000CHA (Kidney), ENCSR000CGW (Spleen), ENCSR966JPL (Spleen), ENCSR000CHB (Liver), ENCSR000CGX (Cerebellum)

Human research participants

Policy information about studies involving human research participants and Sex and Gender in Research.

Reporting on sex and gender	N/A
Population characteristics	N/A
Recruitment	N/A
Ethics oversight	N/A

Note that full information on the approval of the study protocol must also be provided in the manuscript.

Field-specific reporting

Please select the one below that is the best fit for your research. If you are not sure, read the appropriate sections before making your selection.

☒ Life sciences ☐ Behavioural & social sciences ☐ Ecological, evolutionary & environmental sciences

For a reference copy of the document with all sections, see nature.com/documents/nr-reporting-summary-flat.pdf

Life sciences study design

All studies must disclose on these points even when the disclosure is negative.

Sample size	xDBiT multi-organ experiment:<br>A total of three mice was used for the multi-organ experiments. No statistical method was used to predetermine the sample size. The rationale for this sample size was to have to have enough tissue samples to perform two independent xDBiT experiments on a variety of different tissue types. Since all mice were healthy and wild-type a larger sample size was not required for downstream statistical analyses.

Cross-contamination experiment:
A total of 2 mice was used for the spillover experiment. No statistical method was used to predetermine the sample size. Since the rationale behind this experiment was to test the reliability of the xDBiT workflow and no comparisons between biological conditions were made, as few mice as possible were used.

Data exclusions

On sample level:
The pancreas samples were excluded from downstream analysis due to low read counts.

Data filtering:
- Pre-filtering was done in the processing of the raw RNA-seq to remove short reads and reads with no spatial or genomic information.
- To computationally remove cross-contaminations, the background expression level of genes was measured based on ST spots without underlying tissue. Subsequently, only genes with an expression level higher than twice the standard deviation of the mean background signal were used for downstream analyses.

Replication

xDBiT multi-organ experiment:
Per xDBiT run:
- 2 sections of kidney, spleen, heart and pancreas or cerebellum.
- 1 section of liver. Since xDBiT allows the measurement of 9 sections in parallel, it did not allow more than one replicate for the fifth organ. Due to its high homogeneity, we chose to measure only one replicate of the liver sample.
- We chose this number of replicates to check for both intra and inter-experimental variations by Pearson correlation analysis.

Cross-contamination experiment:
- 4 sections per liver sample. Center well free. Due to the high experimental costs we did this experiment only once.

Randomization

No experimental groups were present in this study.

Blinding

Since there were no experimental groups, blinding was not necessary. All mice were treated the same.

Reporting for specific materials, systems and methods

We require information from authors about some types of materials, experimental systems and methods used in many studies. Here, indicate whether each material, system or method listed is relevant to your study. If you are not sure if a list item applies to your research, read the appropriate section before selecting a response.

Materials & experimental systems

n/a	Involved in the study	
☐	☒	Antibodies
☒	☐	Eukaryotic cell lines
☒	☐	Palaeontology and archaeology
☐	☒	Animals and other organisms
☒	☐	Clinical data
☒	☐	Dual use research of concern

Methods

n/a	Involved in the study	
☒	☐	ChIP-seq
☒	☐	Flow cytometry
☒	☐	MRI-based neuroimaging

Antibodies

Antibodies used

anti-CD31 primary antibody (Thermo Fisher, PA5-16301; dilution: 1:50), AF555 secondary antibody (Invitrogen, A-31572; dilution: 1:500), anti-BSA antibody (Invitrogen, A11133; final concentration: 80 µg/mL)

Validation

All antibodies have been validated by the manufacturer.

Details from manufacturer's website:
- anti-CD31 antibody: This antibody was verified by Relative expression to ensure that the antibody binds to the antigen stated. Purification by Antigen affinity chromatography.
- AF555 secondary antibody: These donkey anti-rabbit IgG (H+L)whole secondary antibodies have been affinity-purified and show minimum cross-reactivity. Cross-adsorption or pre-adsorption is a purification step to increase specificity of the antibody resulting in higher sensitivity and less background staining. The secondary antibody solution is passed through a column matrix containing immobilized serum proteins from potentially cross-reactive species. Only the nonspecific-binding secondary antibodies are captured in the column, and the highly specific secondaries flow through. Cross adsorption against Against bovine, chicken, goat, guinea pig, hamster, horse, human, mouse, rat, and sheep serum.
- anti-BSA antibody: Anti-bovine serum albumin (anti-BSA) antibody is a highly purified rabbit IgG fraction that can be used for the detection of BSA in tracer studies or in protease assays. This antibody demonstrates high specificity for BSA and little or no crossreactivity to other serum proteins.

Animals and other research organisms

Policy information about studies involving animals; ARRIVE guidelines recommended for reporting animal research, and Sex and Gender in Research

Laboratory animals	Wild-type C57BL/6J male mice. Age: 3-4 months.
Wild animals	The study did not involve wild animals.
Reporting on sex	This study did only involve male animals and did not aim to find sex-dependent differences.
Field-collected samples	This study did not involve field collected samples.
Ethics oversight	Animal experiments were carried out in compliance with the German Animal Protection Act and with the approved guidelines of the Society of Laboratory Animals (GV-SOLAS). All animal used within this study were kept at the HMGU Core Facility Laboratory Animal Services (CF-LAS), Neuherberg, Germany. All procedures were carried out in compliance with German Animal Welfare Legislation and the regulations of the Government of Upper Bavaria, Germany. Animal housing was approved according to §11 of the German Animal Welfare Act and performed in accordance with Directive 2010/63/EU.

Note that full information on the approval of the study protocol must also be provided in the manuscript.
